# Solar Flare Detection from Sudden Ionospheric Disturbances in VLF Signals via a CNN–HMM Framework

**DOI:** 10.3390/s26082548

**Published:** 2026-04-21

**Authors:** Yuliyan Velchev, Boncho Bonev, Ilia Iliev, Peter Gallagher, Peter Z. Petkov, Ivaylo Nachev

**Affiliations:** 1 Department of Radio Communications and Video Technology, Faculty of Telecommunications, Technical University of Sofia, 1000 Sofia, Bulgaria; julian_s_velchev@tu-sofia.bg (Y.V.); bbonev@tu-sofia.bg (B.B.); igiliev@tu-sofia.bg (I.I.); ivaylonachev@tu-sofia.bg (I.N.); 2 Astronomy and Astrophysics, Dublin Institute for Advanced Studies, DIAS Headquarters, 10 Burlington Road, D04 C932 Dublin, Ireland; peter.gallagher@dias.ie

**Keywords:** solar flare detection, sudden ionospheric disturbances, VLF, convolutional neural network, hidden Markov model, Viterbi algorithm

## Abstract

In this paper we present a hybrid convolutional neural network–hidden Markov model framework for detecting solar flare events of intensity greater than or equal to M1.0 from very low frequency signals via their induced sudden ionospheric disturbances. The convolutional neural network processes fixed-length windows of raw very low frequency signals and their temporal derivatives to produce probabilistic flare estimates, which serve as emission probabilities for a two-state hidden Markov model. Viterbi decoding enforces temporal consistency, suppressing spurious fluctuations and yielding physically plausible event sequences. The approach is specifically designed to detect the onset-to-peak interval of flare events and, with further development, could operate in real time for early flare warning. The model was trained and evaluated on very low frequency data from the DHO38 transmitter in Germany to a receiver near Birr, Ireland. Sample-level evaluation achieved a balanced accuracy of 0.819 and a Matthews correlation coefficient of 0.529, while event-level detection reached a peak F1-score of 0.558 for moderate-to-strong flares of intensity greater than or equal to C6.0. These results demonstrate automated, physically consistent detection of solar flares based on sudden ionospheric disturbances, indicating the potential of the proposed approach, when combined across multiple receivers, to act as a low-cost complement to satellite-based monitoring.

## 1. Introduction

A solar flare is an intense eruption of electromagnetic radiation originating in the Sun’s atmosphere [1]. These events significantly change the degree of ionization in the Earth’s atmosphere [2]. During a flare, enhanced levels of high-frequency ultraviolet (UV), extreme ultraviolet (EUV), and X-ray radiation are emitted. They cause a rapid increase in electron density in the ionosphere on the sunlit side of the Earth. These effects mostly affect the lowest part of the ionosphere (the D-region), where enhanced electron content causes an increase in radio-wave absorption. As a result, the propagation of radio waves over a wide range of frequencies is disturbed, particularly in the high-frequency (HF) band, where shortwave fadeouts may occur. Impacts may also be observed in satellite-based navigation and communication systems [3].

A solar flare usually lasts from several minutes to several tens of minutes [4], but associated ionospheric effects may continue to exist longer depending on flare intensity. The occurrence rate of solar flares varies with the solar cycle, ranging from less than one event per week during solar minimum to several events per day near solar maximum. In general, weaker flares occur more frequently than stronger ones.

Depending on the peak level of the soft X-ray flux (1Å to 8Å wavelength band) measured on Earth, the solar flares are classified into A, B, C, M, and X categories (Table 1) [5], where each successive class represents a tenfold increase in peak flux. For example, an X-class flare is ten times more intense than an M-class flare. Within each class, events are further subdivided numerically (e.g., M1 to M9, X1 to X9 and higher), providing finer resolution of flare strength.

Timely prediction of solar flares is of particular importance because intense solar storms can cause damage to electronic systems, communication equipment, and electric power infrastructure. In the context of radio blackouts and impacts on communication and power systems, M- and X-class flares are generally considered significant. Strong X-class flares produce substantial ionization in the lower ionosphere, particularly in the D-region, leading to enhanced absorption of radio waves.

Sudden ionospheric disturbance (SID) is a phenomenon associated with rapid ionospheric perturbations resulting from enhanced ionization in the D-region caused by solar flare radiation [6,7,8,9,10]. The increase in ionization is primarily driven by solar X-rays and extreme UV radiation reaching the sunlit side of the Earth.

**Figure 1:**
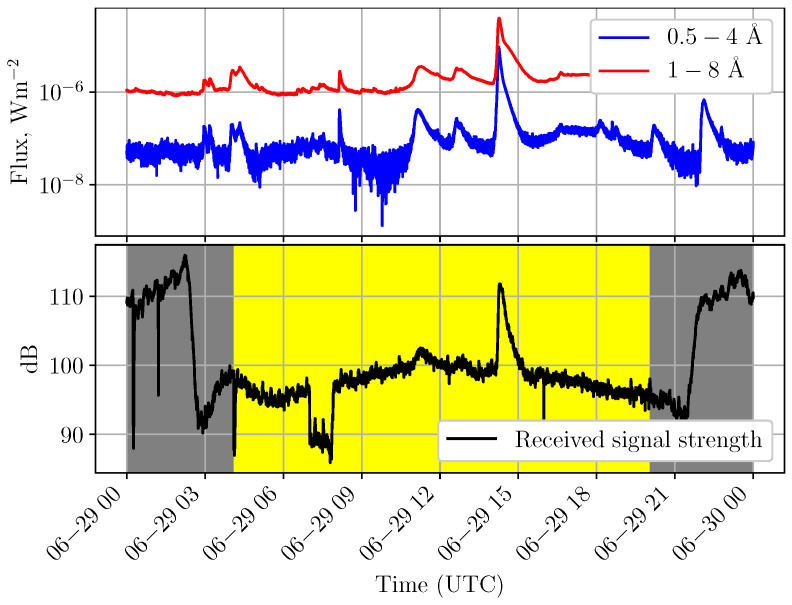
Example of a SID observed in the VLF range on 29 June 2023. The lower panel shows the abrupt increase in received signal strength caused by an M3.8-class solar flare peaking at 14:15, while the upper panel presents the corresponding X-ray flux. The interval during which the propagation path is sunlit is highlighted in yellow.

In narrow-band very low frequency (VLF) radio transmissions, the received signal amplitude often changes significantly during such events [7,11] (Figure 1). Depending on the geometry of the propagation path and local ionospheric conditions, the signal strength may increase or decrease. This abrupt variation in received signal power begins almost immediately, or within at most a few minutes after the onset of the solar flare. The rapid response is due to the fact that VLF waves propagate within the Earth–ionosphere waveguide and are reflected from the lower boundary of the ionosphere, whose effective reflection height decreases as electron density increases in the D-region. The duration of the disturbance is typically longer than the impulsive phase of the flare and is characterized by a gradual recovery of the received signal toward its pre-flare level.

Although solar flares increase ionization throughout all ionospheric regions, the relative enhancement is strongest in the D-region [12]. Under normal daytime conditions, this region is primarily maintained by solar Lyman-α radiation at 121.567 nm, which ionizes nitric oxide [13,14]. During solar flares, however, ionization in the D-region is dominated by soft X-rays with wavelengths shorter than approximately 10 Å, which ionize molecular nitrogen and oxygen. This produces a substantial increase in electron density and significantly modifies radio-wave propagation conditions.

For HF waves, the enhanced D-region ionization increases absorption as the waves traverse this region before being reflected by the E- and F-regions, often leading to shortwave fadeouts. In contrast, VLF waves are reflected near the lower ionosphere (upper D-region or lower E-region). During flare conditions, the effective reflection height decreases, shortening the propagation path within the ionosphere and reducing attenuation in the waveguide. As a result, the amplitude and phase of VLF signals may change markedly. This sensitivity of VLF propagation to D-region ionization can be used for continuous monitoring of VLF transmissions from communication and navigation facilities worldwide for the detection of SIDs and the indirect observation of solar flare activity.

Accurately detecting flare-induced SIDs from VLF signals is challenging due to path-dependent variations. Purely neural network models capture complex signal features but may produce temporally inconsistent predictions, while recurrent neural networks also struggle with rapid transitions and do not enforce physically plausible state sequences. To address these limitations, we adopt a hybrid convolutional neural network–hidden Markov model (CNN–HMM) framework: the CNN provides probabilistic SID estimates for short VLF windows, and the HMM with Viterbi decoding enforces temporal consistency, suppressing spurious fluctuations and yielding physically meaningful event sequences. This sample-based approach allows for online detection of solar flares ≥ M1, which are the minimum intensity class that can cause significant ionospheric disturbances affecting radio communications, navigation, and power systems.

The main contributions of this work can be summarized as follows:

- End-to-end SID detection from raw VLF time series: Unlike previous approaches that rely on hand-crafted or VLF-derived features [15], the proposed framework operates directly on raw amplitude measurements, enabling automated feature extraction through a CNN.
- Integration of temporal modeling via HMM: By combining CNN predictions with a HMM and Viterbi decoding, the approach enforces temporal consistency, suppresses isolated false positives, and improves event-level detection of solar flares;
- HMM transition priors are derived from flare statistics from 2016 to 2025, while the CNN is trained on VLF data from August 2023 to December 2025.
- Evaluation at sample and event levels: Performance is quantified using multiple metrics (precision, recall, F1-score, balanced accuracy, and Matthews correlation coefficient) on over 500,000 quality-controlled time samples, providing a comprehensive assessment of the detector’s capabilities.
- Baseline for single-path SID monitoring: The results demonstrate that reliable flare detection is possible from a single VLF propagation path, establishing a low-cost, ground-based complement to satellite flare monitoring and a foundation for future multi-path integration and phase analysis.

## 2. Related Work

Numerous studies have demonstrated that flare-induced X-ray and EUV enhancements modify the reflection height and conductivity of the Earth–ionosphere waveguide, resulting in measurable amplitude and phase perturbations along VLF propagation paths [16,17]. While these techniques provide valuable physical insight and are computationally efficient, they often require manual parameter tuning and may suffer from reduced sensitivity under noisy propagation conditions or during overlapping ionospheric disturbances. These limitations highlight the need for more adaptive and automated detection frameworks capable of capturing complex temporal patterns in VLF responses to solar flares. Observational and modeling investigations have quantified D-region ionization dynamics and provided theoretical frameworks for interpreting flare-induced signal variations [18,19]. These methods typically compare real-time measurements with climatological averages or reference curves to detect deviations exceeding predefined limits. Statistical techniques, including correlation analysis with Geostationary Operational Environmental Satellite (GOES) X-ray flux measurements and superposed epoch analysis, have also been widely applied to confirm flare-induced perturbations. Complementary experimental analyses of specific transmitter–receiver paths further confirmed the sensitivity of VLF signals to solar X-ray flux enhancements [20]. More recent investigations emphasize the importance of automated and robust flare detection techniques to improve real-time space weather monitoring capabilities [21]. In parallel, remote sensing-based approaches increasingly incorporate data-driven methodologies for detecting ionospheric disturbances [22]. Despite these advances, most VLF-based flare detection methods use thresholding or they rely on manual interpretation, which limits their robustness under noisy propagation conditions and motivates the development of hybrid machine learning frameworks capable of capturing both spectral features and temporal evolution of flare-induced perturbations.

A significant body of work in solar flare research has explored image-based detection and classification using both traditional computer vision and machine learning techniques. Early efforts in automatic flare detection from full-disk solar images demonstrated that structural properties and brightness enhancements can be used to identify flare events in optical and EUV imagery [23]. More recently, advanced instruments such as the Solar Disk Imager onboard the Advanced Space-based Solar Observatory have enabled dedicated automated detection pipelines that utilize intensity changes within segmented macropixel regions to identify and track flares in ultraviolet full-disk images [24]. These methods focus on identifying local intensity enhancements and managing simultaneous or temporally close flare events, showing high detection efficiency compared with GOES X-ray catalogs [24]. Beyond detection algorithms tailored to a specific imager, CNNs [25] have been broadly applied for solar flare classification and prediction from magnetic field data and multi-wavelength imagery. For example, CNN-based models trained on magnetogram and EUV image datasets have achieved competitive performance in binary flare forecasting and multi-class classification tasks [26,27], highlighting the potential of deep learning for extracting discriminative flare signatures from high-dimensional image data. In parallel, statistical and machine learning approaches grounded in pattern recognition and image features, such as tolerance near sets, have also been proposed to classify flares from observational images using GPU-accelerated computing frameworks [28]. These works illustrate a transition from heuristic image analysis toward data-driven and deep learning-based image recognition methods for solar flare detection, which motivates integrating such capabilities with complementary time series methods.

Beyond image recognition and direct VLF perturbation techniques, a range of complementary methods have been proposed to detect or characterize solar flare impacts on the ionosphere and associated radio signals. Recent work has examined the nowcasting capability of VLF measurements across day–night mixed propagation paths, showing that amplitude and phase maxima correlate with X-ray flux even when conventional day-only paths are not available [29]. Data-driven anomaly detection using autoencoder networks on VLF series has also been explored as an unsupervised way to identify flare effects in amplitude variations [30]. Alternative HF radio techniques, such as signal-to-noise ratio analysis from ionospheric sounders, enable detection of ionospheric absorption changes induced by flare events, particularly at mid-latitudes [31]. Global Ionospheric Flare Detection Systems have been developed to provide near-real-time monitoring of flare X-ray events by leveraging distributed VLF receivers and correlating their responses with enhanced ionization [32]. Earlier approaches utilize GPS-derived total electron content (TEC) data to monitor flare-induced ionospheric ionization changes, demonstrating that TEC variations correspond with major flare episodes [33]. Additionally, studies using Digisonde and Doppler sounding methods have evaluated delays between solar flare radiation and ionospheric signal changes, providing insight into temporal response characteristics of both electron density and geomagnetic field perturbations [34]. Other research has employed remote sensing of TEC and scintillations to assess the influence of simultaneous ionospheric and geomagnetic perturbations during flare events, highlighting how electromagnetic and plasma parameters jointly affect radio propagation [35]. These diverse approaches collectively broaden our understanding of solar flare impacts on terrestrial and space-based measurement systems, underscoring the importance of hybrid detection frameworks that can integrate multiple signal types and temporal scales. An autoencoder-based neural network is applied on VLF-derived features for SID detection [15]. In the cited work, the authors reported relatively moderate detection capability and high false positive rates. Machine learning models have been widely applied to flare forecasting using solar magnetograms or EUV imagery. Depending on the dataset, forecast window, and evaluation protocol, these approaches typically report true skill statistics (TSS) in the range of approximately 0.4 to 0.7 for M-class flare prediction [36,37]. However, these methods focus on forecasting solar activity from solar observations rather than detecting ionospheric responses in ground-based measurements.

Only a very limited number of studies have addressed automated SID detection from VLF measurements using modern machine learning techniques. Most operational ground-based approaches rely on amplitude or phase thresholding relative to quiet-day baselines, which, while computationally simple, are sensitive to path-specific variability and often exhibit elevated false positive rates under disturbed propagation conditions.

## 3. Materials and Methods

### 3.1. Pipeline of the Proposed Approach

**Figure 2:**
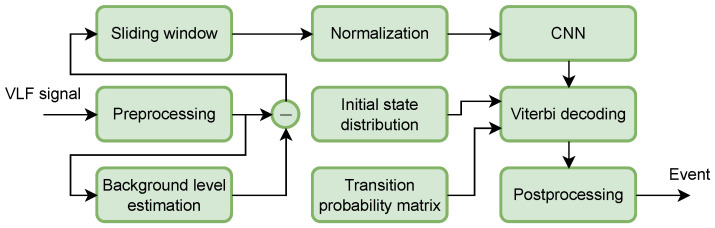
Block diagram of the proposed pipeline for solar flare detection using SID in VLF signals.

Figure 2 illustrates the overall pipeline of the proposed approach for solar flare detection using the SID effect observed in VLF signals.

First, the acquired VLF signal undergoes preprocessing. The raw amplitude is converted to a logarithmic scale. Since the sampling rate may differ between recordings, the signal is resampled to a unified one. A low-pass filter is then applied to suppress high-frequency noise and unwanted fluctuations. Next, the background level of the signal is estimated and subtracted to emphasize perturbations associated with solar flare activity. A sliding window mechanism is subsequently applied, where each input consists of 24 sequential samples, including the current and preceding observations. The window advances with a step of one sample, ensuring fine temporal resolution. The resulting windowed signal is normalized before being fed into a one-dimensional CNN. The 1D CNN produces probabilistic outputs, which are interpreted as emission probabilities for a HMM [38]. These probabilities are combined with an estimated transition probability matrix and an initial state distribution vector within a Viterbi decoding framework. The Viterbi algorithm determines the most likely sequence of hidden states corresponding to flare and non-flare conditions. Finally, the decoded state sequence is subjected to a postprocessing stage, where the detected event boundaries are refined based on characteristic features of the signal. This step compensates for limitations in accurately determining the event offset.

The approach operates in a fully sample-based manner, which is particularly advantageous for early event detection, as it enables fine-grained temporal analysis and prompt identification of the onset of solar flare activity.

### 3.2. VLF Radio Path and Dataset

**Figure 3:**
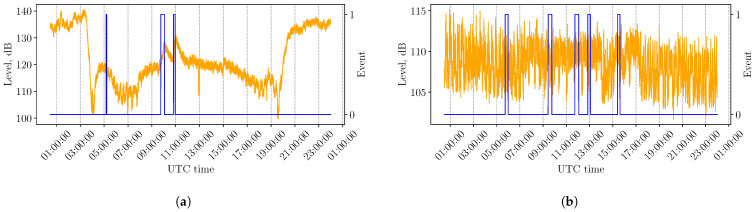
Examples of VLF signals from the SuperSid dataset [39] for the DHO38 transmitter. The received VLF signal level, in logarithmic scale, is shown in orange, whereas solar flare events (begin-to-peak) with intensity ≥ M1.0 are shown in blue. (**a**) An example with good signal quality on 29 April 2025. (**b**) An example with poor signal quality on 10 July 2024.

Regarding the detection of solar flares based on ionospheric reflections, the SuperSid system (which serves to monitor the space weather) has been used [39]. It consists of inexpensive radio receivers that track many VLF transmissions over the world. Since the geographical location has strong impact on the atmospheric ionization, we tailor our model to a specific transmitter–receiver path. We have selected DHO38 transmitter used by the German Navy. It is located near Rhauderfehn, Germany, with 53.08° latitude and 7.61° longitude. It transmits at 23.4 kHz with a power of up to 800 kW. The chosen receiver is located at latitude 53.10° and longitude −7.92° (Dunsink observatory, DIAS, Birr, Ireland). We selected only records that contain at least one solar flare with an intensity greater than or equal to M1.0. This selection strategy is motivated by the rarity of solar flares: for effective training and testing, the limiting factor is not the abundance of quiet periods but the availability of days with actual flare events, ensuring that the model learns meaningful flare-related signal patterns. We used records spanning from August 2023 to December 2025, as earlier data are highly fragmented and could not be reliably used for training or testing. From the selected period, we manually assessed signal quality and retained only the days with good-quality VLF recordings, discarding those with poor quality. Bad-quality signals are characterized by significantly lower overall levels and the presence of unusual repeating patterns with periods ranging from several minutes to approximately half an hour. The origin of these repeating patterns could not be conclusively identified (see Figure 3b). A representative good-quality example is shown in Figure 3a.

For the DHO38 transmitter, we observed significantly reduced signal levels for periods of about one to two hours in the early morning (Figure 3a—approximately from 5:30 to 7:30). It is presumed that the transmitter undergoes scheduled maintenance during this interval. When estimating the background level, we select parameters that minimize the influence of these short signal dropouts on the background calculation.

We further restrict the analysis to fragments of VLF signals for which the cosine of the solar zenith angle, evaluated at the great-circle midpoint between the transmitter and receiver, satisfies

(1) $$\cos\left(\theta \right)\geq 0.25,$$

as a significant SID response in VLF is expected only under this condition [40].

Only fragments with good signal quality were used for training and evaluation. Periods of degraded signal quality were identified automatically using a detection procedure based on a median-filtered local baseline. Low power level fragments were detected using a sliding window of 30 s; if the median signal level within the window dropped by more than 8 dB below the corresponding median baseline level, the segment was marked as low level. To ensure robust exclusion of disturbed periods, the detected intervals were extended by 10 min on both sides using morphological dilation. All samples belonging to these low-quality intervals were removed from subsequent analysis.

The solar flare onset, peak, and end times were obtained from the SpaceWeatherLive database [41], which compiles and visualizes solar flare data. SpaceWeatherLive itself relies on primary observations from the GOES series operated by the National Oceanic and Atmospheric Administration, which provides continuous X-ray flux measurements in the 1 to 8 Å and 0.5 to 4 Å bands. These X-ray measurements are the standard for classifying solar flares (C, M, X classes) and determining their timing. The flare timing used in this study is based on the GOES X-ray flux data, with a temporal resolution of one minute. These records were used as the ground truth for event identification and temporal annotation in our analysis.

The resulting dataset is randomly partitioned into approximately three equal subsets for three-fold cross-validation during training and evaluation. To preserve temporal structure, the partitioning is performed at the level of daily sequences rather than individual time points. In each fold, the model is trained on two-thirds of the sequences, while the remaining one-third is used for testing. This process is repeated three times, ensuring that each sequence is used exactly once for testing. The test outputs from all folds are then aggregated to assess overall model performance.

### 3.3. Signal Preprocessing, Background Removal, and Normalization

The VLF recordings used exhibit varying sampling intervals, with 5 s being the most typical, whereas the ground truth flare timings from SpaceWeatherLive (GOES X-ray data) have a temporal resolution of one minute. To achieve better temporal alignment and maintain good time resolution for analyzing the SID response, we resampled the VLF data to a uniform 10 s interval using Fourier-based interpolation.

After resampling, the VLF signals were smoothed using an eighth-order low-pass Butterworth filter to suppress high-frequency noise while retaining the slower variations associated with SID responses. The filter was applied with a cutoff frequency corresponding to a 2 min period using zero-phase filtering.

To estimate the slowly varying background of the VLF signals, we applied a robust two-step procedure designed to preserve long-term trends while removing short-term fluctuations. First, the signal was smoothed using a median filter with a kernel size corresponding to approximately 3 h, which effectively suppresses transient spikes and short-term noise. To further refine the background and reduce small-scale variability, the median-filtered signal was then processed with a Savitzky–Golay filter using a window of half the median kernel size and a third-order polynomial. Edge effects were handled by symmetric padding prior to filtering.

To improve the training stability of the CNN, all features were normalized using z-score normalization (standard scaling). Each feature was centered by subtracting its mean and scaled by dividing by its standard deviation, resulting in transformed features with zero mean and unit variance.

### 3.4. Convolutional Neural Network Model

The convolutional neural network is designed as a one-dimensional temporal feature extractor and probabilistic state estimator operating on fixed-length VLF windows. Each input consists of two channels—the raw VLF signal and its first-order temporal derivative. This allows the model to capture both absolute amplitude levels and rapid variations characteristic of flare-induced disturbances.

**Figure 4:**
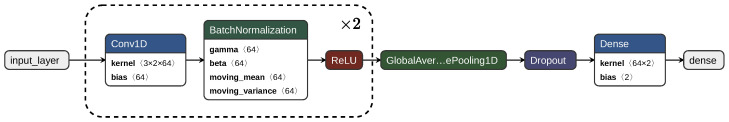
One-dimensional CNN architecture used for flare detection.

The architecture comprises two identical one-dimensional convolutional layers, each with 64 filters and a kernel size of 3, using causal padding to preserve strict temporal alignment (Figure 4). Each convolutional layer is followed by batch normalization and a rectified linear activation function. The extracted feature maps are aggregated using global average pooling, providing a compact and noise-robust representation. A dropout layer with a rate of 0.3 is applied before the final fully connected layer. It outputs unnormalized logits for the two classes (flare and non-flare). Posterior state probabilities are obtained via a softmax transformation and interpreted as emission probabilities for the hidden Markov model.

Class weights are applied during training to mitigate the strong class imbalance. The network is trained for 20 epochs using the Adam optimizer with a learning rate of $10^{-3}$. A variant with three convolutional layers was also evaluated and yielded similar performance; the two-layer configuration was therefore retained for lower computational complexity.

### 3.5. Hidden Markov Model and Viterbi Decoding

The temporal dynamics of SIDs are modeled using a HMM with two discrete states, $S_{t}\in \left\{0,1\right\}$, representing the absence or presence of a flare-induced disturbance. Let $A=[a_{ij}]$ denote the state transition matrix,

(2) $$a_{ij}=P\left(S_{t}=j|S_{t-1}=i\right),$$

which encodes physically plausible transitions between states. The initial state distribution is assumed to be uniform $\pi =\left[\begin{array}{cc} 0.5 & 0.5 \end{array}\right]$, which is sufficient because the Viterbi recursion quickly converges to a stable state sequence.

The emission probabilities are provided by the CNN. For each time step *t*, the emission likelihood is

(3) $$p\left(x_{t}|S_{t}=s\right)\propto P\left(S_{t}=s|x_{t}\right),$$

where $x_{t}\in R^{24}$ denotes the 24-sample input window of VLF signal and derivative features. Given the CNN-derived emissions ${\left\{p_{t}\right\}}_{t=1}^{T}$ and the transition matrix A, the most probable sequence of hidden states $\hat{S}=\left\{{\hat{S}}_{1},\ldots ,{\hat{S}}_{T}\right\}$ is obtained using the Viterbi algorithm [42], which maximizes the joint probability:

(4) $$\hat{S}=\arg\max_{S_{1},\ldots ,S_{T}}\pi_{S_{1}}\;p\left(x_{1}|S_{1}\right)\prod_{t=2}^{T}a_{S_{t-1}S_{t}}\;p\left(x_{t}|S_{t}\right).$$

For numerical stability, the Viterbi recursion is implemented in the logarithmic domain. Defining the log-path score

(5) $$\delta_{t}\left(j\right)=\max_{S_{1},\ldots ,S_{t-1}}\log p\left(S_{1},\ldots ,S_{t-1},S_{t}=j,x_{1:t}\right),$$

the recursion is written as

(6) $$\delta_{t}\left(j\right)=\max_{i}\left[\delta_{t-1}\left(i\right)+\log a_{ij}\right]+\log p\left(x_{t}|S_{t}=j\right),\;t=2,\ldots ,T,$$

with initialization

(7) $$\delta_{1}\left(j\right)=\log\pi_{j}+\log p\left(x_{1}|S_{1}=j\right),$$

and backpointers

(8) $$\psi_{t}\left(j\right)=\arg\max_{i}\left[\delta_{t-1}\left(i\right)+\log a_{ij}\right].$$

After processing the full sequence, the optimal final state is

(9) $${\hat{S}}_{T}=\arg\max_{j}\delta_{T}\left(j\right),$$

and the complete sequence $\hat{S}$ is recovered by backtracking through $\psi_{t}\left(j\right)$.

The transition probability matrix A is derived from ground-truth flare data spanning 2016 to 2025, capturing variations in solar activity. These statistics only define the HMM’s prior dynamics. In contrast, the CNN is trained on VLF recordings from August 2023 to December 2025. Therefore, this study does not claim full solar-cycle validation; rather, the HMM prior is based on flare data covering varying solar activity levels.

### 3.6. Postprocessing

Since the proposed CNN–HMM framework is optimized for onset-to-peak detection of solar flares with intensity greater than or equal to M1, we observed a systematic latency in the estimated offset (peak) time. Specifically, the probabilistic classifier tends to maintain elevated posterior probabilities after the true flare maximum, which propagates through the Viterbi decoding and results in a delayed transition from the positive to the negative state. To compensate for this effect, a dedicated postprocessing stage was introduced to refine the peak localization. The adjustment is based on the physical characteristics of the VLF signal, where the SID response exhibits a well-defined amplitude extremum at the flare maximum. After the HMM identifies a candidate flare segment, the offset is corrected by aligning it with the local extremum (maximum perturbation) of the VLF amplitude within a constrained temporal window around the decoded transition. In addition, very short events with duration shorter than 30 s are discarded to reduce spurious detections.

## 4. Results

### 4.1. Evaluation Strategy

The sample-level evaluation assesses classification performance at the temporal resolution of individual VLF time samples. Each time sample is treated as a separate binary classification instance, where the positive class corresponds to solar flares of intensity M1.0 or higher.

Samples corresponding to periods of degraded VLF signal quality were excluded prior to evaluation. After this quality control step, a total of 537,909 time samples were retained for performance assessment. This quality control applies to both sample-level and event-level evaluations, ensuring that only reliable VLF data contribute to the reported metrics.

From the confusion matrix—comprising true positives (TP), false positives (FP), false negatives (FN), and true negatives (TN)—standard performance metrics are computed. The precision (positive predictive value, PPV) quantifies the reliability of positive predictions

(10) $$PPV=\frac{TP}{TP+FP}.$$

The recall (true positive rate, TPR) measures detection sensitivity

(11) $$TPR=\frac{TP}{TP+FN}.$$

The F1-score, defined as the harmonic mean of precision and recall, provides a single measure balancing both

(12) $$F1=\frac{2\;\cdot \;PPV\;\cdot \;TPR}{PPV+TPR}.$$

In addition, specificity (true negative rate, TNR)

(13) $$TNR=\frac{TN}{TN+FP},$$

false positive rate (FPR)

(14) $$FPR=\frac{FP}{FP+TN},$$

false negative rate (FNR)

(15) $$FNR=\frac{FN}{FN+TP},$$

overall accuracy (ACC)

(16) $$ACC=\frac{TP+TN}{TP+TN+FP+FN},$$

and balanced accuracy (BA)

(17) $$BA=\frac{TPR+TNR}{2}$$

are also reported. The Matthews correlation coefficient (MCC)

(18) $$MCC=\frac{TP\;\cdot \;TN-FP\;\cdot \;FN}{\sqrt{\left(TP+FP)(TP+FN)(TN+FP)(TN+FN\right)}}$$

is particularly informative under strong class imbalance, as it incorporates all four entries of the confusion matrix and provides a balanced measure even when one class dominates.

While sample-level metrics quantify point-wise classification performance, they do not fully reflect the operational objective of detecting complete flare events. Therefore, an event-level evaluation is also performed. A detected flare is considered a true positive event if its predicted onset-to-peak interval overlaps with an actual flare event in the reference dataset (ground truth). A false positive event corresponds to a predicted flare segment without a matching reference event, whereas a false negative event denotes a missed reference flare. As for sample-level metrics, only events occurring within high-quality VLF periods are included in this evaluation. Event-level performance is quantified using precision, recall, and F1-score.

### 4.2. Detection Results

The sample-level performance is evaluated for both the standalone CNN and the proposed CNN–HMM framework (Table 2). For the CNN, probabilistic predictions are converted to class labels using a threshold of 0.5, while CNN–HMM uses Viterbi decoding. Both models achieve a good balance between sensitivity and specificity.

Compared to the standalone CNN, the CNN–HMM framework improves precision and F1-score while maintaining a similar recall. The FPR is reduced from 0.043 to 0.025, and overall reliability is enhanced, as reflected by a significant increase in MCC from 0.442 to 0.529. The positive-class recall remains moderate, capturing most flare onsets, while performance on the negative class remains high.

**Figure 5:**
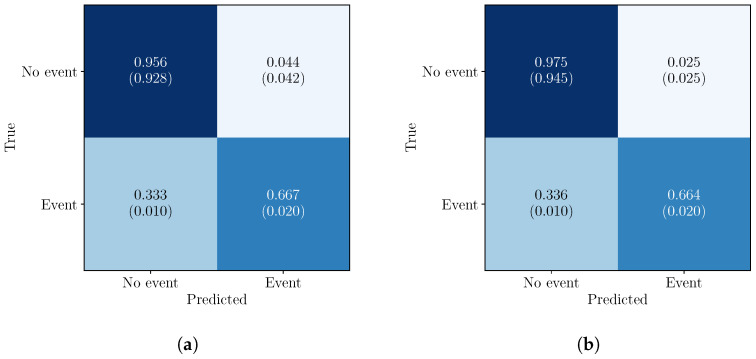
Sample-level confusion matrices for CNN only (**a**) and the CNN–HMM framework (**b**). The upper values correspond to row normalization, whereas the values in brackets indicate full normalization. The total number of samples is 537,909.

The row-normalized version of the confusion matrices (Figure 5) shows high true negative rates (97.5% for CNN–HMM vs. 95.7% for CNN) and comparable flare detection performance (approximately 66%). The fully normalized matrices highlight the class imbalance, with correctly classified non-flare samples dominating (92.8% for CNN vs. 94.5% for CNN–HMM). False positives are notably higher for the CNN, while false negatives remain similar between the models.

Overall, the CNN–HMM model improves reliability by reducing false positives. Accordingly, subsequent analysis focuses on the CNN–HMM framework.

The model is trained to detect ≥M1.0 events flares, but since weaker C-class events can produce detectable VLF signatures, we also evaluated the performance at lower thresholds (≥C1.0, ≥C2.0, etc.). Event-level performance for CNN-HMM framework across different minimum flare intensity thresholds is shown in Table 3.

The F1-score reaches a maximum of 0.558 at ≥C6.0. Precision increases as the threshold decreases, while recall declines, reflecting that weaker C-class flares are less consistently associated with observable SID signals. These results demonstrate that the model not only captures M-class events and above, for which it was trained, but also effectively identifies moderate-to-strong C-class flares.

**Figure 6:**
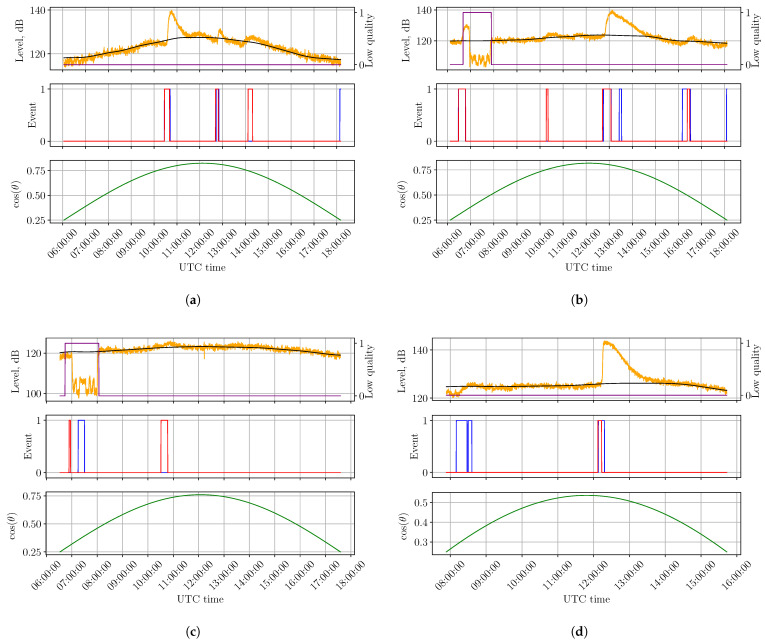
Examples of solar flare detections obtained with the proposed CNN–HMM framework. For each example, the top panel shows the VLF signal (orange), the estimated background level (black), and intervals of low signal quality (purple). The middle panel presents the true ≥M1.0 flare events (blue) and the predicted ones (red). The bottom panel shows cos(θ). (**a**) False positive around 14:10 on 28 July 2024 caused by a rapid background-level change. (**b**) False negative around 13:30 on 31 July 2024 due to SID masking by a preceding flare. (**c**) A false positive occurred around 10:30 on 18 August 2024, associated with a local peak in the X-ray flux corresponding to C7.46. However, it was not reported as a solar flare in [41], as the flux enhancement did not exhibit a sufficiently rapid rise. (**d**) False negative around 08:30 on 03 October 2024 caused by reduced flare sensitivity at low cos(θ).

The proposed CNN–HMM framework has several limitations:

- False positive detections may occur during rapid background-level variations (Figure 6a), which are not fully tracked by the background estimation module;
- False negatives can arise when a flare-induced SID is masked by a preceding event (Figure 6b), reflecting the intrinsic inertia of the ionosphere and thus representing a fundamental physical limitation;
- Ambiguities in flare definition, particularly regarding the required rise time of the X-ray flux, may lead to discrepancies between detected events and reported flare catalogs, producing false positives for slowly rising but intense flux enhancements (Figure 6c);
- The sensitivity decreases when the Sun is close to the horizon (low cos(θ)), where the SID effect is weaker (Figure 6d). This limitation could be mitigated by employing multiple transmitters and receivers to improve spatial coverage and robustness.

## 5. Discussion

The Sudden Ionospheric Disturbance (SID) effect is a complex and highly variable geophysical phenomenon influenced by solar X-ray flux, ionospheric state, diurnal variability, and propagation path characteristics. This intrinsic variability makes solar flare detection from a single VLF propagation path particularly challenging, as weak or moderate flares may induce perturbations comparable to ambient noise levels. Nevertheless, the reported sample- and event-level results demonstrate that real-time or near real-time solar flare detection is feasible using the proposed CNN–HMM framework.

Direct quantitative comparison with existing SID detection or flare forecasting studies is limited by differences in datasets and evaluation protocols. Many flare forecasting studies report skill scores such as the TSS over multi-hour prediction windows, whereas the present work evaluates instantaneous sample-level classification and event-level detection using precision, recall, F1-score, balanced accuracy, and the Matthews correlation coefficient. In addition, this study is based on a single quality-controlled VLF propagation path, in contrast to multi-station VLF networks or solar-imagery-based datasets commonly used in other approaches. Within this particularly demanding single-path setting, the achieved event-level peak F1-score of 0.558 at ≥C6.0 and sample-level MCC of 0.529 indicate competitive discrimination capability.

Compared to the autoencoder-based SID detection method in [15], which relies on engineered VLF-derived features and frame-wise classification in a learned latent space, the proposed CNN–HMM framework operates directly on raw time-series samples and incorporates explicit probabilistic temporal modeling via HMM decoding. Although an exact quantitative comparison is not possible due to differing datasets and evaluation procedures, the results suggest that structured temporal decoding contributes to improved false positive control and more consistent event-level detection.

Notably, although the model was trained using ≥M1.0 flares as the positive class, performance is higher for moderate C-class events and above. This suggests that the CNN learns features related to the magnitude of ionospheric perturbations rather than strictly replicating the GOES flare classification threshold. Future work could explore training with higher-intensity thresholds (e.g., ≥M5 or ≥M6), where SID responses are more pronounced and potentially more distinguishable from background variability.

The present evaluation is limited to a single VLF propagation path, which constrains spatial coverage and robustness. Incorporating multiple transmitter–receiver paths would enable the fusion of independent detections, thereby improving confidence, reducing false positives, and enhancing geographic applicability. Such a multi-path strategy would also mitigate path-specific noise and propagation effects, increasing overall detection reliability.

From a practical perspective, the CNN–HMM framework with Viterbi decoding provides a low-cost, ground-based complement to satellite-based flare detection systems and direct solar flux measurements. Although performance metrics remain moderate, they validate the underlying concept and demonstrate that SID-based flare detection is operationally feasible even under single-path conditions.

## 6. Conclusions

This study presents a CNN–HMM framework for detecting solar flares from VLF radio signal perturbations. By operating directly on raw time-series samples and incorporating probabilistic temporal modeling via Viterbi decoding, the proposed approach enables consistent event-level detection from a single quality-controlled VLF propagation path. The peak event-level F1-score of 0.558 at ≥C6.0 and sample-level MCC of 0.529 indicate that the framework can reliably discriminate flare-induced perturbations from background variability, despite the inherent challenges of weak or moderate SID signatures.

Compared to existing SID detection methods, the proposed CNN–HMM approach offers explicit temporal sequence modeling, contributing to improved false positive control and more consistent event-level detection.

The present work is limited to a single VLF path, which constrains spatial coverage and sensitivity. With further development, including multi-path integration and leveraging both amplitude and phase information from the VLF signals, this approach could evolve into a complementary tool for space weather monitoring, enabling timely alerts for M-class or stronger flares at a fraction of the cost associated with dedicated space-based instrumentation.

## Figures and Tables

**Table 1:** Solar flares classification.

Class	Peak Flux, W m^−2^
A	<1 × 10^−7^
B	1 × 10^−7^–1 × 10^−6^
C	1 × 10^−6^–1 × 10^−5^
M	1 × 10^−5^–1 × 10^−4^
X	>1 × 10^−4^

**Table 2:** Sample-level performance for CNN only and the CNN–HMM framework.

Metric	CNN	CNN–HMM
Positive-Class Metrics		
PPV (Precision)	0.324	0.449
TPR (Recall)	0.667	0.664
F1	0.436	0.536
Negative-Class Metrics		
TNR (Specificity)	0.957	0.975
FPR	0.043	0.025
FNR	0.333	0.336
Overall Metrics		
ACC (Accuracy)	0.948	0.965
BA (Balanced Accuracy)	0.812	0.819
MCC	0.442	0.529

**Table 3:** Event-level performance for CNN-HMM framework at different minimum flare intensity thresholds. The highest F1-score is highlighted in bold.

Event Threshold	Events	PPV (Precision)	TPR (Recall)	F1
≥M1.0	211	0.354	0.797	0.491
≥C9.0	234	0.388	0.784	0.519
≥C8.0	264	0.419	0.752	0.538
≥C7.0	308	0.459	0.704	0.556
≥C6.0	370	0.497	0.635	**0.558**
≥C5.0	442	0.520	0.557	0.538
≥C4.0	526	0.545	0.489	0.515
≥C3.0	617	0.566	0.433	0.491
≥C2.0	706	0.577	0.386	0.463
≥C1.0	749	0.579	0.366	0.448

## Data Availability

The datasets used for experimental part in this study is publicly available at https://vlf.ap.dias.ie/ (accessed on 15 April 2026). The source codes are available from the corresponding author upon reasonable request.

## Abbreviations

The following abbreviations are used in this manuscript:

CNN	Convolutional neural network
GOES	Geostationary operational environmental satellite
HF	High frequency
EUV	Extreme ultraviolet
HMM	Hidden Markov model
LF	Low frequency
SID	Sudden ionospheric disturbance
TEC	Total electron content
TSS	True skill statistics
UV	Ultraviolet
VLF	Very low frequency
